# Application of a Multiplex Quantitative PCR to Assess Prevalence and Intensity Of Intestinal Parasite Infections in a Controlled Clinical Trial

**DOI:** 10.1371/journal.pntd.0004380

**Published:** 2016-01-28

**Authors:** Stacey Llewellyn, Tawin Inpankaew, Susana Vaz Nery, Darren J. Gray, Jaco J. Verweij, Archie C. A. Clements, Santina J. Gomes, Rebecca Traub, James S. McCarthy

**Affiliations:** 1 Clinical Tropical Medicine Laboratory, QIMR Berghofer Medical Research Institute, Herston, Queensland, Australia; 2 Department of Veterinary Disease Biology, Faculty of Health and Medical Science, University of Copenhagen, Copenhagen, Denmark; Department of Parasitology, Faculty of Veterinary Medicine, Kasetsart University, Bangkok, Thailand; 3 Research School of Population Health, College of Medicine, Biology and Environment, The Australian National University, Canberra, Australian Capital Territory, Australia; 4 Molecular Parasitology Laboratory, QIMR Berghofer Medical Research Institute, Herston, Queensland, Australia; 5 School of Public Health, University of Queensland, Herston, Queensland, Australia; 6 Laboratory for Medical Microbiology and Immunology, St Elisabeth Hospital, Tilburg, the Netherlands; 7 Laboratorio Nacional da Saúde, Ministério da Saúde, Dili, Timor-Leste; 8 Faculty of Veterinary and Agricultural Sciences, University of Melbourne, Parkville, Victoria, Australia; 9 School of Medicine, University of Queensland, Queensland, Australia; George Washington University, UNITED STATES

## Abstract

**Background:**

Accurate quantitative assessment of infection with soil transmitted helminths and protozoa is key to the interpretation of epidemiologic studies of these parasites, as well as for monitoring large scale treatment efficacy and effectiveness studies. As morbidity and transmission of helminth infections are directly related to both the prevalence and intensity of infection, there is particular need for improved techniques for assessment of infection intensity for both purposes. The current study aimed to evaluate two multiplex PCR assays to determine prevalence and intensity of intestinal parasite infections, and compare them to standard microscopy.

**Methodology/Principal Findings:**

Faecal samples were collected from a total of 680 people, originating from rural communities in Timor-Leste (467 samples) and Cambodia (213 samples). DNA was extracted from stool samples and subject to two multiplex real-time PCR reactions the first targeting: *Necator americanus*, *Ancylostoma* spp., *Ascaris* spp., and *Trichuris trichiura*; and the second *Entamoeba histolytica*, *Cryptosporidium* spp., *Giardia*. *duodenalis*, and *Strongyloides stercoralis*. Samples were also subject to sodium nitrate flotation for identification and quantification of STH eggs, and zinc sulphate centrifugal flotation for detection of protozoan parasites. Higher parasite prevalence was detected by multiplex PCR (hookworms 2.9 times higher, *Ascaris* 1.2, *Giardia* 1.6, along with superior polyparasitism detection with this effect magnified as the number of parasites present increased (one: 40.2% vs. 38.1%, two: 30.9% vs. 12.9%, three: 7.6% vs. 0.4%, four: 0.4% vs. 0%). Although, all STH positive samples were low intensity infections by microscopy as defined by WHO guidelines the DNA-load detected by multiplex PCR suggested higher intensity infections.

**Conclusions/Significance:**

Multiplex PCR, in addition to superior sensitivity, enabled more accurate determination of infection intensity for *Ascaris*, hookworms and *Giardia* compared to microscopy, especially in samples exhibiting polyparasitism. The superior performance of multiplex PCR to detect polyparasitism and more accurately determine infection intensity suggests that it is a more appropriate technique for use in epidemiologic studies and for monitoring large-scale intervention trials.

## Author Summary

Gastrointestinal parasites including soil-transmitted helminths cause considerable morbidity worldwide, especially in resource-poor communities. Large-scale epidemiologic and treatment efficacy studies are regularly undertaken to determine the optimum ways to reduce or eliminate parasites from endemic communities, thereby reducing the burden of disease. Accurate and sensitive tests for detection of soil transmitted helminths and protozoa are of great importance to the success of such trials. Increasingly recognised is the importance of accurately determine the infection intensity, as morbidity and transmission pressure of helminth infections are directly related this and not just to prevalence. A vast majority of studies use standard microscopy methods which, although well accepted, may not be as accurate as more recently developed molecular techniques such as multiplex PCR. Therefore, there is need for further evaluation of multiplex PCR techniques and their ability to detect infections and provide infection intensity data. In the current study real-time PCR showed a higher sensitivity for the detection of intestinal helminths and protozoa especially in cases of mixed infections as well as more accurate determination of infection intensity compared to microscopy.

## Introduction

Gastrointestinal parasites including soil-transmitted helminths (STH) cause considerable morbidity worldwide, especially in resource-poor communities. The chronic effects on health are predominately attributed to the burden of disease rather than mortality [1]. The majority of the global burden is considered due to the five main STH–*Ascaris lumbricoides*, hookworms (*Necator americanus* and *Ancylostoma* spp.), *Trichuris trichiura* and *Strongyloides stercoralis* [2–4]; with a significant burden also due to protozoan infections. Polyparasitism is especially widespread, and the impact of this is likely to be more severe than single parasite infections [5–7]. Reliable diagnostic techniques suitable for accurate and sensitive identification of parasites in terms of infection intensity are essential in order to determine effectiveness of disease control programs. This is because morbidity and transmission pressure of helminth infections are directly related to both the prevalence and intensity of infection [8,9].

Several microscopy-based techniques are available and widely used for the identification and quantification of STH eggs. The Kato-Katz (KK) thick smear technique, originally developed for diagnosis of schistosomiasis [10], is currently the most widely used microscopic technique, and is considered the gold standard by the World Health Organization (WHO) for assessing both prevalence and intensity of infection in helminth control programmes [11]. A major drawback of the KK is that multiple samples with multiple slides per sample are required to be examined over several days to reach high levels of sensitivity and quantitative accuracy, especially in light infections [12]. Moreover, immediate and skilled processing is required to reduce chance of false-negative results, particularly for hookworms, due to fast clearance on slides [7,13]. Alternative methods for microscopic diagnosis of STH include using concentration steps, such as formalin-ether sedimentation and flotation techniques such as McMaster [14], simple sodium nitrate methods [15], FLOTAC [16] or mini-FLOTAC [17]. These have some advantages including increased sensitivity over KK. For example, in a recent study sodium nitrate methods proved superior for detecting low egg burdens in samples compared to quadruple KK smears and resulted in higher EPG [15]. Comparisons of KK to FLOTAC also showed equivalent [18,19] or superior [20,21] sensitivity of FLOTAC techniques. These flotation based techniques have drawbacks in terms of being labour intensive, having poor reproducibility owing to operator error, and for most tests requiring centrifugation steps [16,19]. In addition, microscopic-based techniques lack the ability to assign species-level identification of helminth eggs (e.g. those of hookworms, and *Ascaris*). Diagnosis of *S*. *stercoralis* is particularly challenging, as only a small number of larvae are released in stool regardless of infection intensity, with Baermann sedimentation or agar plate culture methods thought to provide greatest specificity and sensitivity [22,23]. Serological diagnostic methods are available for STH but their use is limited due to poor specificity in endemic areas [24].

Stained faecal smears and faecal concentration methods allow for diagnosis of protozoa. However, these too have their limitations with regards to poor sensitivity and the inability to differentiate protozoan parasite stages to a species level. For example, diagnosis of *E*. *histolytica* infections by microscopy misses 40% of infections [25]; and it is not possible to visually differentiate pathogenic *E*. *histolytica* from non-pathogenic *Entamoeba dispar*. Thus, only *E*. *histolytica* specific stool antigen detection tests are approved for diagnostic use by the WHO [26], although shown to have poor sensitivity compared to PCR-based methods [27,28]. More efficient coproantigen capture enzyme linked immunosorbent assay (ELISA) based assays can be used for diagnosis of *Cryptosporidium* and *Giardia*, however there have been reports of false-positive and false-negative results [29].

Polymerase chain reaction (PCR)-based techniques are assuming a dominant place in modern diagnostic microbiology. For STH diagnostics they have been shown to be more sensitive than microscopy, particularly at low infection intensities [11,30]. For detection of protozoal infections, PCR-based techniques have shown significantly higher sensitivity compared to microscopy and/or immunodiagnostic techniques [31–37]. Adapting PCR assays to multiplex real-time platforms enables simultaneous detection of multiple parasites, thereby minimizing reagent costs and processing time [12,30,38]. In addition, such assays can be adapted to be quantitative, which is a significant advantage in STH infections where parasite burden rather than presence or absence of infection is a key determinant of morbidity. Multiplex real-time PCR methods for the diagnosis of intestinal parasites [39] have been applied in epidemiological surveys in parasite endemic areas of Ghana, Togo [13], Bangladesh, and recently in the Philippines [40] as well as in hospital samples from symptomatic patients in the Netherlands [38], and Malaysia [41,42]. Despite numerous studies incorporating multiplex PCR methods, very limited data has been published to date with thorough quantitative comparisons between this method and microscopy-based techniques.

The aim of the current study was to evaluate the use of two multiplex PCRs for the detection and quantification of intestinal protozoa and helminths as tools to support large scale epidemiologic and treatment efficacy studies. This method was compared with results obtained with sodium nitrate flotation and zinc sulphate centrifugation for determining prevalence and intensity of intestinal parasite infections in villages in Timor-Leste and Cambodia. A specific aim was to evaluate qPCR as a method for determining infection intensity, an important parameter in both epidemiologic and anthelmintic efficacy studies.

## Methods

### Sample Collection

Samples were sourced from two separate parasitic surveys. Single faecal samples from 467 individuals enrolled in the WASH for WORMS interventional trial in Timor-Leste (Registered with the Australian New Zealand Clinical Trials Registry; Trial registration: ACTRN12614000680662)[43] and from a separate study of 213 individuals enrolled in a cross sectional study of intestinal parasites in northern Cambodia [44]. A single faecal sample per individual was collected within a maximum of 12 hours of defecation and divided into two aliquots and stored separately. One sample in 5% w/v potassium dichromate solution for PCR analysis, and the other sample in 10% formalin for microscopy. Samples from Timor were transported at room temperature to Queensland Berghofer Institute of Medical Research for extraction (QIMRBerghofer) and PCR and Timor-Leste National Lab for microscopy. Cambodian samples were transported at room temperature to the University of Queensland (UQ) Gatton campus for microscopy and DNA extraction, and DNA transported on ice to QIMR Berghofer for PCR.

### Microscopy

All faecal samples were examined microscopically and enumerated for *Ascaris*, hookworms, and *Trichuris* eggs using a simple sodium nitrate flotation as previously described [45]; and for the presence of protozoa cysts and oocysts using zinc sulphate centrifugal flotation [35]. Full methods available in S1 Methods. Specific parasitologic diagnosis of *S*. *stercoralis* infection was not undertaken due to resource limitations that precluded agar plate culture.

### DNA Extraction

Samples stored in 5% potassium dichromate were first subject to centrifugation at 2,000 g for 3 min, followed by removal of the preservative supernatant. Samples were re-suspended to 50 ml with phosphate buffered saline (1 X PBS) and the centrifugation repeated. Supernatant was again decanted off, and the sample pellet was subsequently stored at 4°C for up to a month.

DNA extraction was performed using the Powersoil DNA Isolation Kit (Mo Bio, Carlsbad, CA USA). Minor modifications were made to the manufacturer’s protocol following optimization with *Trichuris vulpis* eggs. Prior to extraction, samples were spiked with a known quantity of a positive control PCR target, namely a plasmid containing equine herpes virus (EHV) insert from the glycoprotein B gene [46]. Full DNA extraction protocol is available in S1 Methods.

### Multiplex PCR

**Fig 1:**
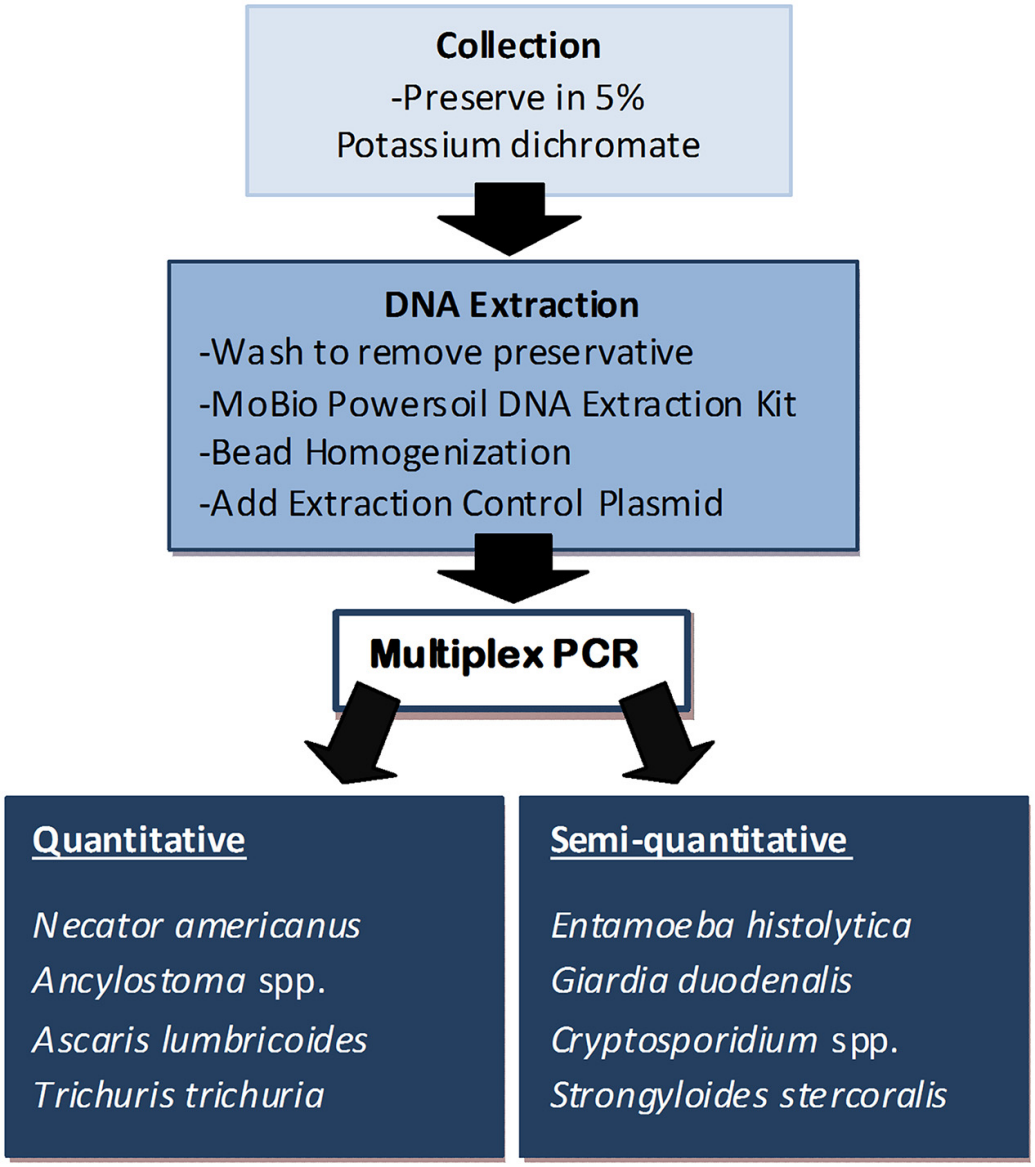
Flow chart of sample processing for multiplex PCR detection of intestinal parasites.

Extracted DNA was run in two pentaplex real-time PCR reactions. The first was a quantitative assay for *N*. *americanus*, *Ancylostoma* spp. (*A*. *duodenale*, *A*. *ceylanicum*), *Ascaris* spp., *T*. *Trichiura* and EHV; the second was a semi-quantitative assay for *E*. *histolytica*, *Cryptosporidium* spp., *G*. *duodenalis*, *S*. *stercoralis* and EHV [12,13,24,34,42,47]. Details of the primers and probes are listed in Tables 1 and 2. However an alternate *S*. *stercoralis* forward primer was used after optimization of this assay with *S*. *stercoralis* positive DNA samples from the Northern Territory (Australia) (Deborah Holt—Personal communication). The Rotor-Gene 6000 (Qiagen, Melbourne, VIC AUS) was used for all PCR assays, with reactions set up for both PCR reactions as previously described [30] with minor modifications. Assay protocol, optimisation and preparation of PCR controls is available in S1 Methods. A sample processing summary is displayed in Fig 1.

**Table 1:** Quantitative multiplex PCR set up overview.

Target	Oligonucletide Sequence 5'—3'		Product Size	Gene Target	Final Conc. nM	GenBank Accession #	Source
*Necator americanus*	Forward	CTGTTTGTCGAACGGTACTTGC	101bp	ITS2	200	AJ001599.1	[22]
	Reverse	ATAACAGCGTGCACATGTTGC			200		
	Probe	FAM- CTGTACTACGCATTGTATAC—MGBNFQ			100		
*Ancylostoma* spp.	Forward	GAATGACAGCAAACTCGTTGTTG	71bp	ITS1	100	EU344797.1	[22]
	Reverse	ATACTAGCCACTGCCGAAACGT			100		
	Probe	VIC- ATCGTTTACCGACTTTAG—MGBNFQ			100		
*Ascaris* spp.	Forward	GTAATAGCAGTCGGCGGTTTCTT	88bp	ITS1	60	AB571301.1	[49]
	Reverse	GCCCAACATGCCACCTATTC			60		
	Probe	ROX -TTGGCGGACAATTGCATGCGAT- IBRQ			100		
*Trichuris trichuria*	Forward	TCCGAACGGCGGATCA	56bp	ITS1	60	FM991956.1	[21]
	Reverse	CTCGAGTGTCACGTCGTCCTT			60		
	Probe	CY5.5 -TTGGCTCGTAGGTCGTT- BHQ-2			100		
Equine Herpes Virus	Forward	GATGACACTAGCGACTTCGA	81bp	gB	40	M26171.1	[48]
	Reverse	CAGGGCAGAAACCATAGACA			40		
	Probe	CY5/FAM—TTTCGCGTGCCTCCTCCAG—IBRQ/ IBFQ			100		

**Table 2:** Semi-quantitative multiplex PCR set up overview.

Target	Oligonucletide Sequence 5'—3'		Product Size	Gene Target	Final Conc. nM	GenBank Accession #	Source
*Entamoeba histolytica*	Forward	AACAGTAATAGTTTCTTTGGTTAGTAAAA	135bp	SSU rRNA	200	X75434.1	[37]
	Reverse	CTTAGAATGTCATTTCTCAATTCAT			200		
	Probe	ROX—ATTAGTACAAAATGGCCAATTCATTCA—IBRQ			80		
*Giardia duodenalis*	Forward	GACGGCTCAGGACAACGGTT	63bp	SSU rRNA	200	M54878.1	[45]
	Reverse	TTGCCAGCGGTGTCCG			200		
	Probe	CY5—CCCGCGGCGGTCCCTGCTAG—IBRQ			100		
*Cryptosporidium* spp.	Forward	CAAATTGATACCGTTTGTCCTTCTG	150bp	COWP	300	AF248743.1	[35]
	Reverse	GGCATGTCGATTCTAATTCAGCT			300		
	Probe	HEX—TGCCATACATTGTTGTCCTGACAAATTGAAT—IBFQ			75		
*Strongyloides* spp.	Forward	GGGCCGGACACTATAAGGAT*	471bp	SSU rRNA	100	AF279916.2	[29]
	Reverse	TGCCTCTGGATATTGCTCAGTTC			100		
	Probe	CY5.5—ACACACCGGCCGTCGCTGC—BHQ-2			100		
Equine Herpes Virus	Forward	GATGACACTAGCGACTTCGA	81bp	gB	40	M26171.1	[48]
	Reverse	CAGGGCAGAAACCATAGACA			40		
	Probe	CY5/FAM—TTTCGCGTGCCTCCTCCAG—IBRQ/ IBFQ			100		

*Altered from the original published primer [29]

### Controls

A Ct cut-off of 31 for *Ascaris* was established based on the limit of detection on a previously published conventional PCR, to ensure reproducibility of results [48]. The limit of detection of all other assays in terms of the maximum Ct-value considered to be positive was set at 35. All PCR assays were validated at independent laboratories (Queensland Medical Laboratory, Australia; The Task Force for Global Health, USA; St. Vincent’s Hospital, Sydney), and additional positive *E*. *histolytica* (n = 2) and *E*. *dispar* (n = 1) control samples tested to ensure PCR specificity for the pathogenic species, *E*. *histolytica*. Further confirmation of *Entamoeba* spp. presence in all controls was provided by a genus specific conventional PCR [49].

### PCR Ct to EPG Conversion—Seeding Experiments

To produce a calibration curve to interpolate EPG values from PCR Ct-values a series of seeding experiments were conducted for *Ascaris* spp. and *N*. *americanus* infections. *Ascaris suum* eggs were purchased from Excelsior Sentinel Inc. (Ithaca, NY), supplied in unembryonated form and stored in 5% potassium dichromate at room temperature for shipping. Hookworm eggs were freshly isolated from three *N*. *americanus* infected stool samples, kindly provided by Alex Loukas (James Cook University). *Ascaris* and hookworm eggs were purified separately [50], and multiple 10 μL aliquots counted under a microscope following staining with Lugol’s iodine solution to determine the concentration of eggs in each sample. Purified eggs were pooled and suspended in a total of 5 ml PBS and diluted to produce a range of concentrations of eggs. *Ascaris* eggs were prepared in triplicate in 2 ml screw top tubes in concentrations ranging from 200,000 EPG to 5 EPG, with an additional 200 mg negative control faecal sample added to each sample. Hookworm eggs were similarly prepared but concentrations ranged from 6,000 EPG to 250 EPG, and were only performed in duplicate due to a smaller number of available egg numbers. Both *Ascaris* and hookworm eggs at each of the concentrations were subject to DNA extraction and multiplex PCR as stated previously. PCR Ct-values were converted to intensities based on assumed 100% reaction run efficiency, provided by the Rotorgene Q software (Multiplex PCR Intensity = 10^−0.298^*^Ct +9.81^). This allowed the interpolation of the relation between the log transformed EPG and log transformed PCR intensity to determine a relationship between Ct-values and EPG.

### Statistical Analysis

Kappa statistics were used for comparison of multiplex PCR and microscopy determined prevalence. Analysis was performed using SPSS (IBM Corp.), Excel 2008 (Microsoft), and GraphPad Prism version 6.0 (GraphPad Software Inc).

### Ethical Considerations

The WASH for WORMS interventional trial in Timor-Leste complies with the provisions contained in the National Statement on Ethical Conduct in Human Research and was approved by the University of Queensland Medical Research Ethics Committee (#2011000734), the ANU Human Research Ethics Committee (protocol: 2014/311), and the Timor-Leste Ethics Committee of the Ministry of Health (reference 2011/51). The Cambodia sample collection study protocol [44] was approved by the National Ethics Committee for Health Research, Ministry of Health, Cambodia (NECHR, #192,) Ethics Committee of the Cantons of Basel-Stadt and Baselland (EKBB, #18/12). Written informed consent was obtained for all participants.

## Results

### Multiplex PCR Optimisation

**Fig 2:**
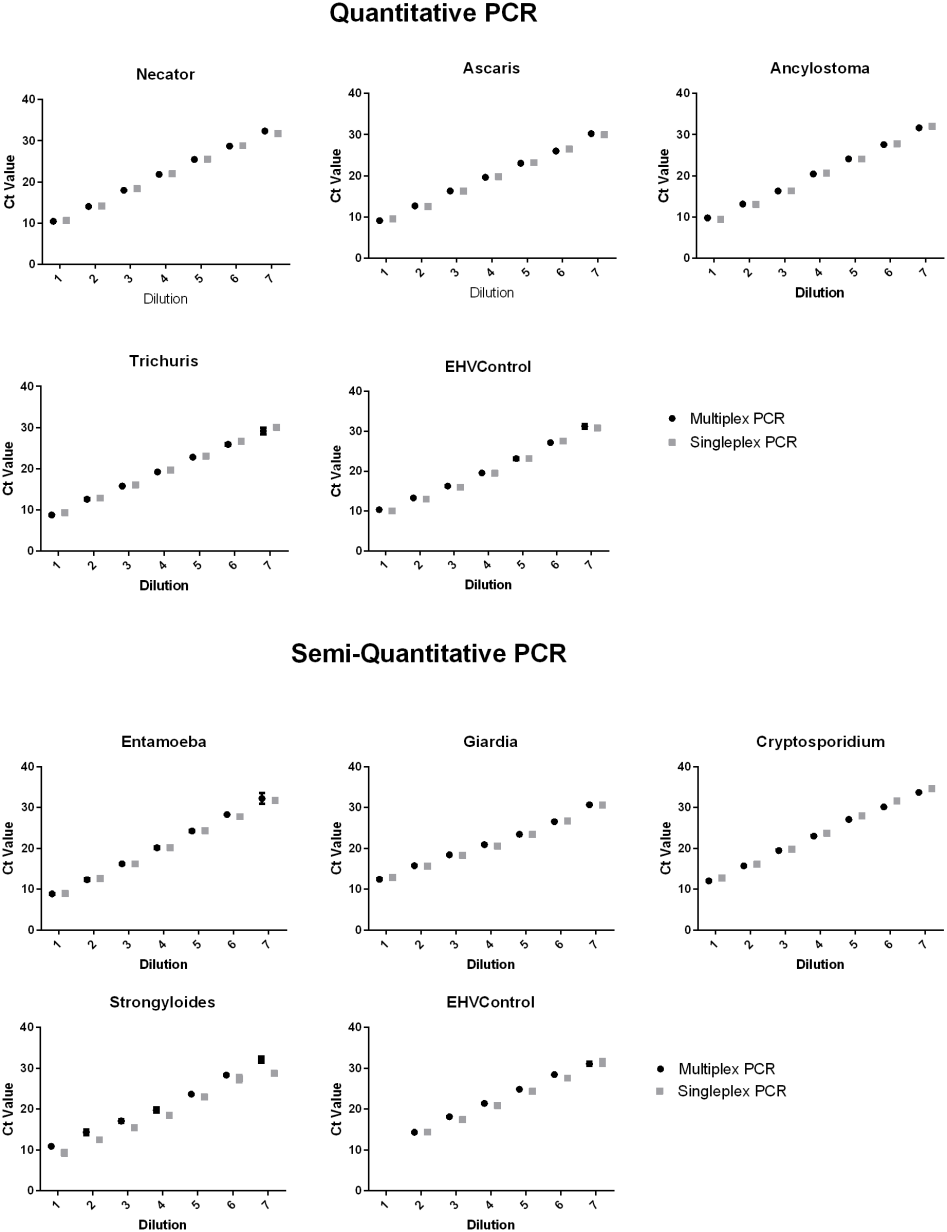
Multiplex to singleplex PCR Ct comparison. Assay optimization to determine effects of multiplex PCR set up on sensitivity and efficiency of PCRs compared to singleplex PCR using plasmid standard curve controls containing all PCR products.

Optimized oligonucleotide concentrations used throughout testing are listed in Table 1. Experimental comparisons of plasmid control dilutions series’ were undertaken between singleplex and multiplex PCR reactions (Fig 2). No or minimal effect on sensitivity and efficiency of one PCR on another was found in each multiplex PCR. Example data of standard EHV Ct-values results is shown in S1 Fig. Field samples excluded due to inhibition of the EHV control were subject to repeat analysis.

### Detection of Single Infections—Diagnostic Performance of PCR vs Microscopy

**Fig 3:**
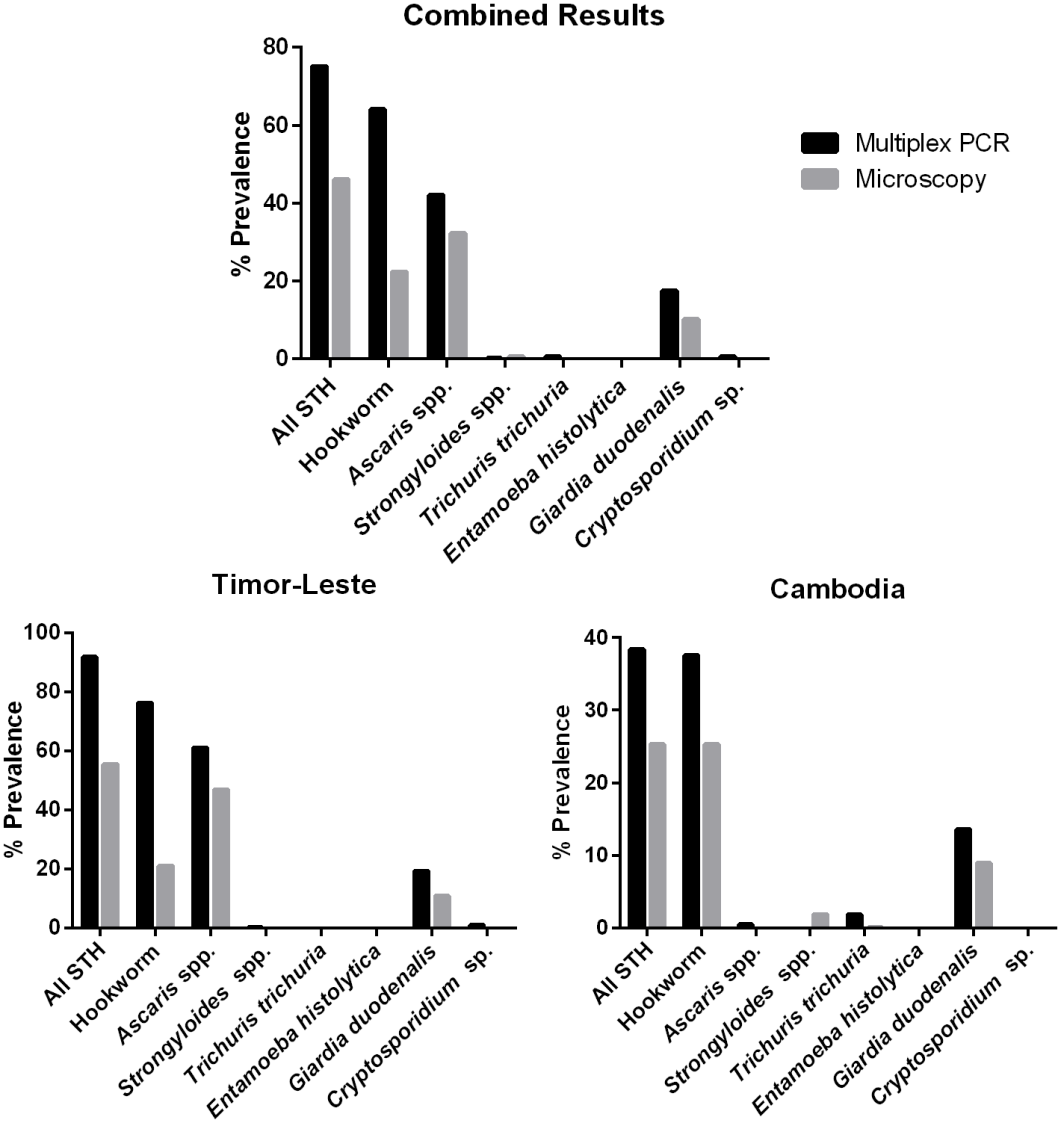
Overall parasite prevalence comparison between multiplex PCR and microscopy. Data presented combined for all 680 study participants, as well as individually for Timor-Leste (467 participants) and Cambodia (213 participants), showing higher recorded percentage prevalence across all target organisms by Multiplex PCR.

Comparison of the prevalence of parasite infection between multiplex PCR and microscopy was made individually for each data set (Fig 3; Table 2; S1 Dataset). The observed multiplex PCR prevalence was consistently higher across nearly all target organisms in both regions studied. Multiplex PCR detected almost three times (2.9, 435/151) the number of hookworm infections, 1.2 (260/219) times more *Ascaris* infections and 1.6 (115/70) times more *Giardia* infections than microscopy at both study sites. *S*. *stercoralis* was however detected in four of the Cambodia microscopy samples, which tested negative in multiplex PCR. The number of samples that tested negative in microscopy was twofold (2.34) higher than the number testing negative in multiplex PCR, indicating that a large proportion of infections were missed by microscopy.

Direct comparisons between diagnostic techniques on individual samples from all regions were undertaken using Kappa agreement statistics (Table 3). Results show good, moderate and fair agreement for *Ascaris*, *Giardia*, and hookworms, respectively. Only target organisms with over 20% prevalence were analysed. For all parasites analysed, multiplex PCR identified a large number of positive samples not detected by microscopy (*Ascaris* 68, Hookworm 299, *Giardia* 69), whilst a small number of microscopy positive samples were not identified as positive by multiplex PCR (*Ascaris* 27, Hookworm 15, *Giardia* 24).

**Table 3:** Multiplex PCR and microscopy parasite prevalence agreement statistics.

	PCR	Microscopy		Total Agreement (%)	Kappa*	SE of Kappa
		POS	NEG			
**Ascaris**	**POS**	192	68	585(86.0)	0.695	0.029
	**NEG**	27	393			
**Hookworm**	**POS**	136	299	366(53.8)	0.201	0.024
	**NEG**	15	230			
**Giardia**	**POS**	46	69	587 (86.3)	0.424	0.049
	**NEG**	24	541			

*Kappa Agreement Level: **K** <0.20 **Poor**; 0.21–0.40 **Fair**; 0.41–0.60 **Moderate**; 0.61–0.80 **Good**; 0.81–1.00 **Very Good**

### Detection of Multiple Infections

**Fig 4:**
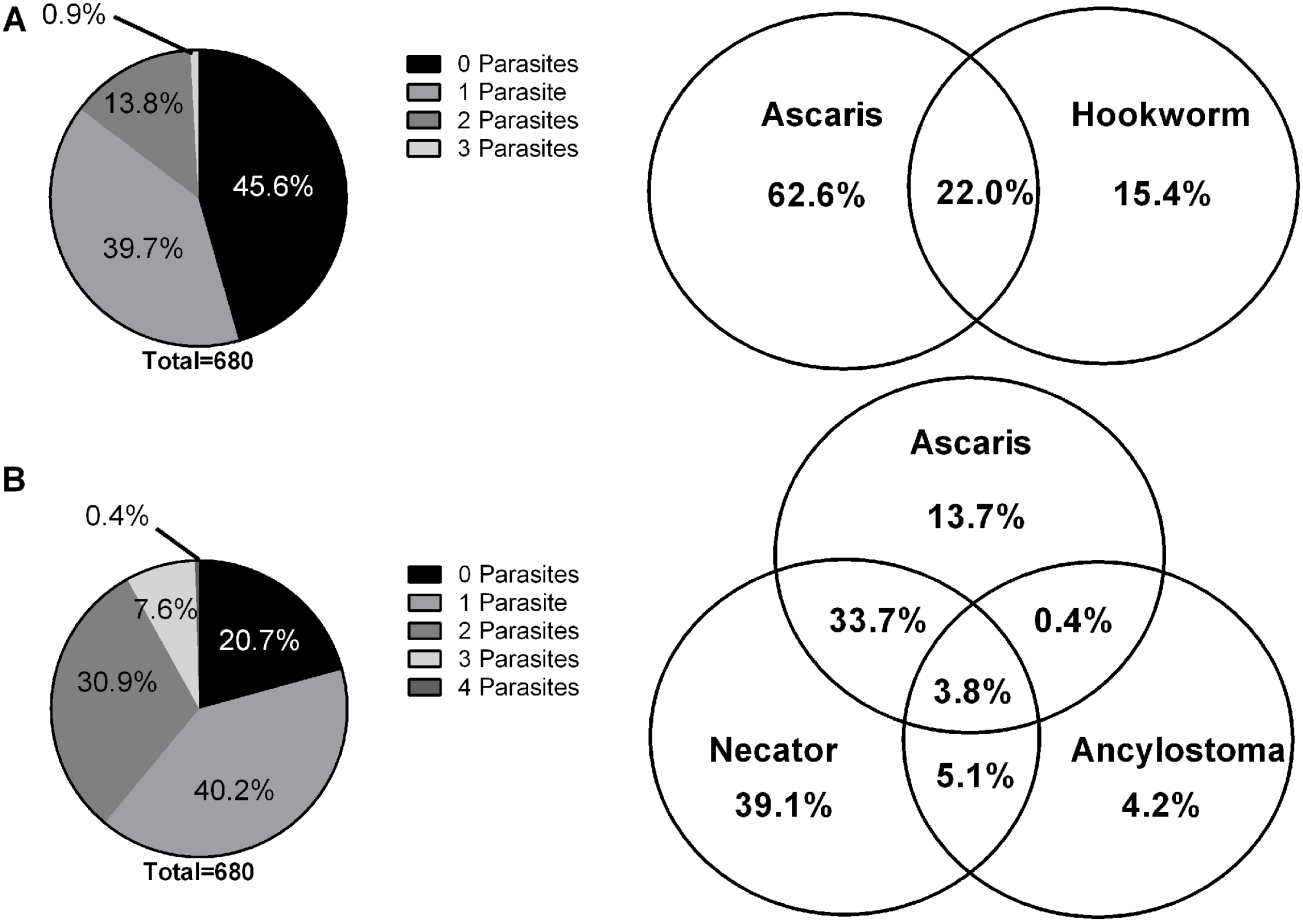
Polyparasitism. Diagrams depict polyparisitism observed in the 680 combined Timor-Leste and Cambodia samples in both **(A) Microscop**y and **(B) Multiplex PCR**. Pie graph depicts total number of parasites per sample and the venn diagram details the specific division of STH coinfections for Microscopy (259 *Ascaris* and/or hookworm positive samples) and multiplex PCR (504 *Ascaris* and/or hookworm positive samples). *^Microscopy unable to differentiate Hookworm species *N*. *americanus* and *Ancylostoma* spp. -considered only as ‘hookworms’ for polyparasitism comparison.^

Along with a higher detection rate of all individual target organisms, the multiplex PCR approach was also superior in terms of sensitivity in detecting samples with multiple infections (Fig 4).

Increased polyparasitism was detected in multiplex PCR in comparison to microscopy, as similar levels of single parasite infections were detected but more than double (2.4 times) the number of dual parasite infections. This trend is further compounded with three parasites, with PCR detecting over 17-fold (17.3) the number of infections than microscopy. An additional three samples were found to harbour four parasites (*Ascaris*, *N*. *americanus*, *Ancylostoma* spp., and *Giardia*), only detected using multiplex PCR. Coinfection with the two genera of hookworms, *N*. *americanus* and *Ancylostoma* spp. that was detected in the multiplex PCR resulted as well in an increased prevalence of polyparasitism.

### Quantitative Results

**Fig 5:**
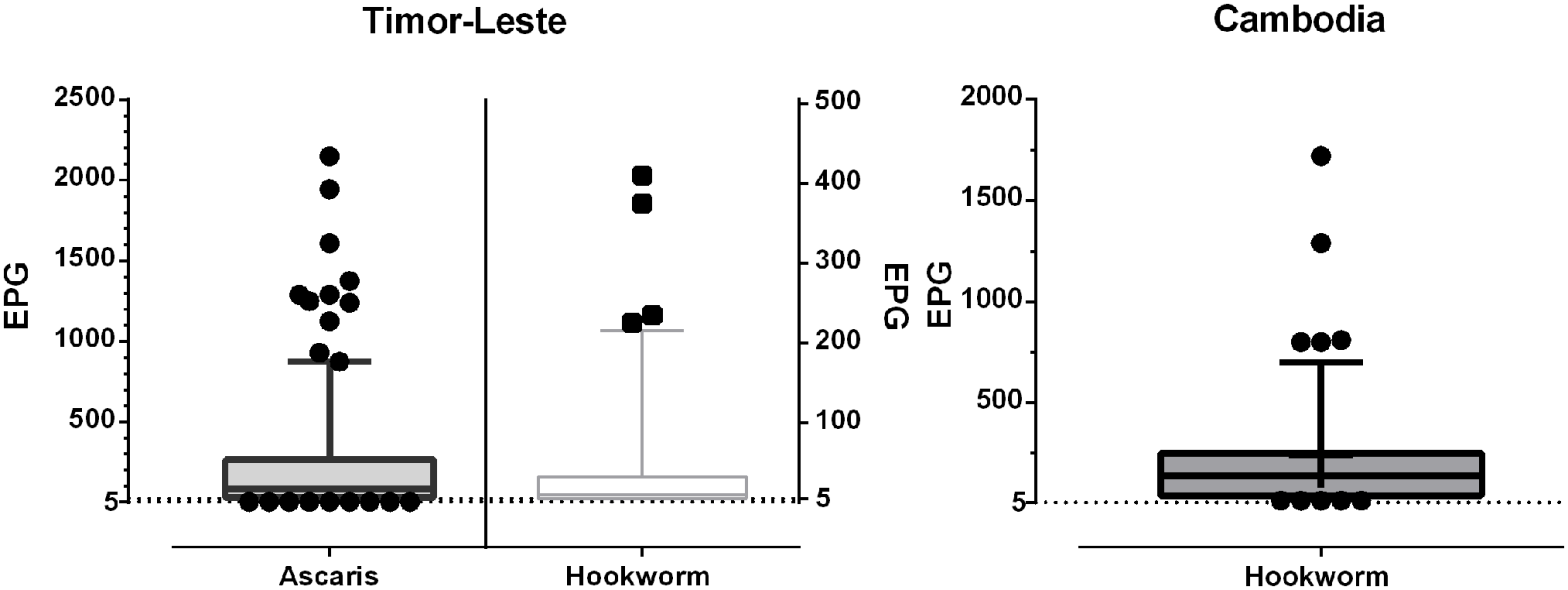
Intensity of infection for *Ascaris* spp. and hookworm positive samples as determined by sodium nitrate flotation. Timor-Leste microscopy produced 219 *Ascaris* positive samples (200 EPG average), 97 hookworm positive samples (40 EPG average). Cambodia microscopy produced 54 hookworm positive samples (60 EPG average).

**Fig 6:**
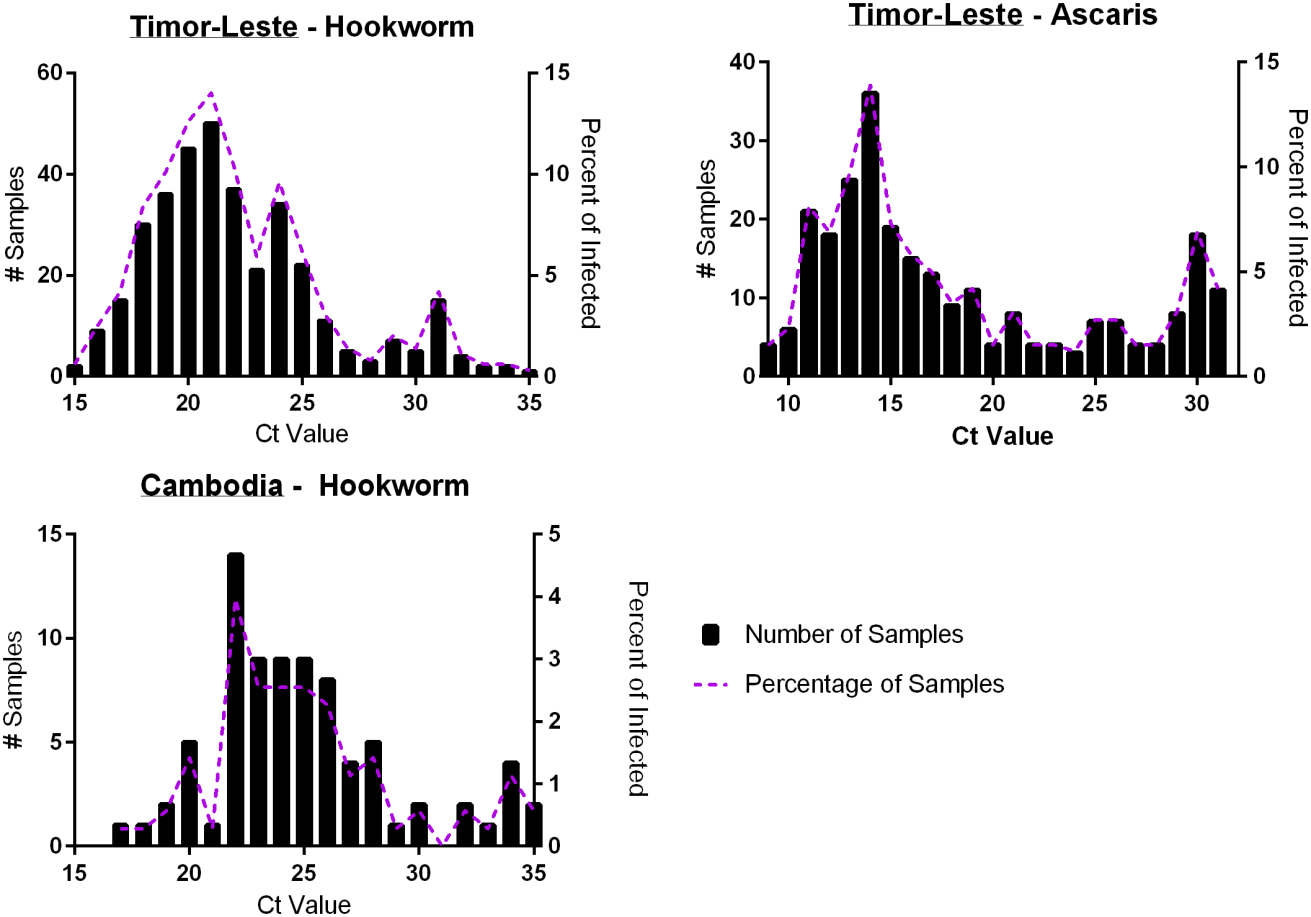
Multiplex PCR Ct-value frequency distribution for hookworm and *Ascaris* spp. positive samples. Graphs show the infection intensity distribution, with lower Ct-values indicating higher infection intensities, presented for Timor-Leste hookworm (353) and *Ascaris* (259), as well as Cambodia Hookworm (80) positive samples. As Ct-values are expressed in a continuous format, values were rounded up to the nearest integer to produce categorical data for frequency analysis.

Infection intensities as determined by microscopy data for nematodes with greatest prevalence is presented in Fig 5. According to WHO guidelines all infections from both study regions for both hookworm and *Ascaris* would be classified as low intensity infections (*Ascaris* < 5,000 EPG; hookworm < 2,000 EPG). The infection intensity frequency distribution of parasites with at least 20% prevalence for Timor-Leste and Cambodia is shown in Fig 6. This Multiplex PCR Ct frequency distribution graph shows the majority of infected individuals harbouring high relative infection burdens (low Ct-values).

The infection intensity of *G*. *duodenale* was similar in both Timor-Leste (Average Ct 24.5; range 16.2–34.3), and Cambodia (Average Ct 23.2; range 16.0–32.0). The few positive *Cryptosporidium sp*. samples in Timor-Leste were all of similar infection intensities (Average Ct 30.9; range 29.0–33.0), whilst the four positive *T*. *trichiura* Cambodian samples all were of low infection intensities (Average Ct 32.7; range 31.5–33.7).

A comparison of quantitative results from both microscopy and multiplex PCR was attempted. However, data are not presented as no statistically relevant relationship was found for either *Ascaris* or hookworms from both study regions. Similarly, no statistical correlation was found between PCR-determined positive samples with low infectivity levels and negative microscopy results, or vice versa.

**Fig 7:**
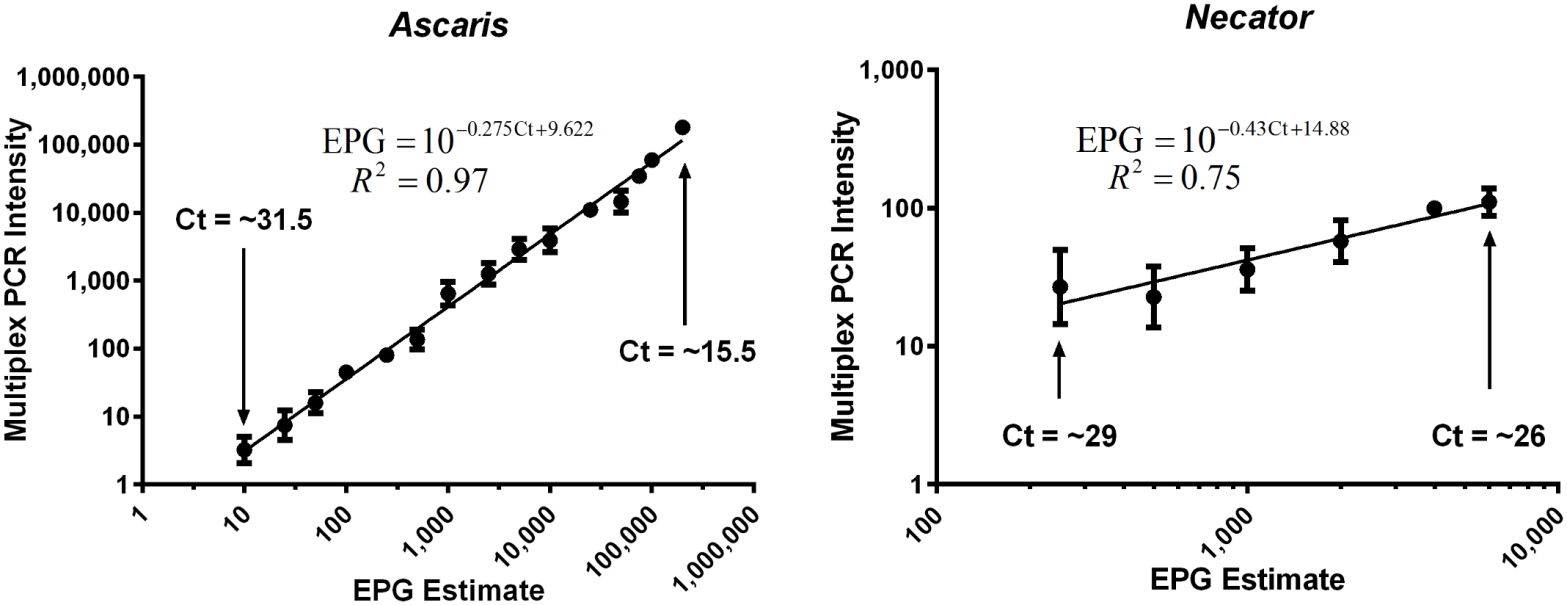
Relationship between EPG and intensity converted PCR Ct-values. Graph shows strong linear relationship (P<0.001) between sodium nitrate flotation determined EPG and Multiplex PCR Intensity upon universal log10 transformation. (95% Confidence Intervals: *Ascaris*- slope 1.028 to 1.089; Y-intercept 0.6405 to -0.4333; X-intercept 0.4205 to 0.5895. Necator—slope 0.3151 to .7344; Y- intercept -0.6150 to 0.7130; X-intercept -2.253 to 0.8413). _* PCR Intensity = 10–0.298*Ct +9.81._

Standard curves obtained from well-defined controls presenting relationship between PCR determined Ct-values (converted to infection intensity) as a measure of EPG derived from standard microscopy practices are shown in Fig 7. The interpolation of Ct-value to EPG derived from this experimental data is also presented within Fig 7, along with the Ct-value range in which this can be reliably used to estimate EPG from multiplex PCR data. A preliminary example of the use of the resulting calibration curve is shown using Timor-Leste field data in S2 Fig.

## Discussion

The present study further demonstrates and validates the suitability of multiplex PCR for detection and quantification of *Ascaris*, hookworms and *Giardia* in stools obtained in large scale surveys. The two multiplex PCRs in *Ascaris*, hookworm and *Giardia* endemic regions of Timor-Leste and Cambodia resulted in significantly higher levels of prevalence compared to microscopy alone [30] [13,36] [51]. Multiplex PCR also showed a greater ability for detection of co-infections, and provided more accurate and reliable infection intensity data; both of key importance to the assessment of disease burden due to the elevated risk of morbidity [52],

A large disparity in the prevalence rates between techniques was noted for the detection of hookworm, *Ascaris* and *Giardia* when compared on a per sample basis. Multiplex PCR in particular detected a large number of positive samples not detected in microscopy; a possible result of the failure of microscopy to detect polyparasitism, present in nearly half (49.1%) of all positives samples by multiplex PCR. A small number of samples were also deemed positive by microscopy but negative in multiplex PCR. This may be due to variation in the dispersion of larvae, eggs and oocytes within the subsamples taken, due to the nonhomogeneous nature of the stool [53], as well as the non-uniform nature of their excretion in stool [30,54], causing variation in both techniques. Differences between techniques may also be due to errors leading to false positive results. Such errors are less likely in multiplex PCR due to rigorous controls, whilst limited controls can be implemented with microscopy, which relies heavily on the technical expertise of the user. This is potentially the case of the four *Strongyloides* positive samples detected only by microscopy, as larvae resemble hatched hookworm larvae. Alternatively further PCR optimization may be required as there have been previous reports of PCR sensitivity issues for detection of *Strongyloides*, as only low levels of larvae are present even in heavy infections [55].

Microscopy sensitivity issues of parasite detection, particularly in samples exhibiting polyparasitism suggest further compounding issues in accurately determining infection intensity levels. Data comparing PCR Ct-values to microscopy determined EPG values have been previously been reported, showing a broad range of Ct-values for each microscopy EPG value; especially for microscopy negative samples [13]. This inability to link the quantitative field data using different techniques despite statistically sound prevalence agreements suggests that multiplex PCR is superior for use in diagnostic testing of STH for survey work where accurate intensity data is required.

This focus on infection intensity is a major strength of the present study when considering disease burden, as STH infection prevalence alone does not provide a measure of potential morbidity, which is related directly to infection intensity [9]. Despite this, the majority of studies have focused on direct prevalence comparisons between techniques, with limited reports on the quantitative abilities of the techniques [30], although some correlation data has been provided [13]. The WHO currently considers prevalence the main measure in STH control programmes, with suggestion of including intensity data only if available, with treatment aims to target medium to heavy STH infections [56]. Providing this more accurate infection intensity data using the multiplex PCR technique may produce data required to establish more effective STH control programmes. The WHO infection intensity estimates may also require re-evaluation with improved intensity data to be useful in PCR-parasite intensity surveys. These estimates were established based using the KK technique, and suggest only low intensity infections when applied to the current study microscopy data (53), whilst multiplex PCR data indicated high intensity infections (low Ct-values) in the majority of individuals.

In the present study, the full relationship between Ct-values and EPG was assessed using standard curves obtained from well-defined controls, with carefully prepared measurements. The feasibility of such an approach has been shown and further indicates that PCR quantification is likely to be more accurate and that additional detailed studies should be undertaken in the future. At this stage insufficient experimental data are available to be able to produce accurate predictions across the whole range of possible Ct-values as the current interpolation is limited to within the microscopy determined EPG range in which it was tested. Additional validation of the *Ascaris* EPG interpolation is required and at higher infection intensities before it can be reliably used with field samples and significant further testing of hookworm samples is essential to produce accurate predictions of EPG. Determining this mathematical relationship between Ct-values, EPG, and by corollary adult worm burden, and assuring its accuracy represents a goal in future research, vital to progress PCR methodologies as the gold standard of intestinal nematode detection. The potential shown here to convert this PCR data to the more widely understood “EPG” format may allow this technique to provide more widely accepted and reportable STH surveillance. However, attempting to enumerate egg counts by PCR without a truly quantitative gold standard for comparison does however, represent a limitation of this work.

Despite proving successful in parasite diagnosis and providing valuable information on infection intensity, there are limitations of multiplex PCR as a diagnostic tool. The use of PCR preservatives such as potassium dichromate and ethanol temporarily arrests further egg development, and are thought to allow accurate quantitative data after many months of storage. However extensive testing into the effect of such preservatives on the genome copy numbers for the target nematodes has not been performed but is crucial for accurate interpolation to an egg count. Further compounding the issue is the ability to relate nematode egg counts to DNA intensity, when copies of DNA increase once eggs have embryonated. Literature does indicate that once embryonated the ITS1 gene target of the PCR assays remains at a constant level [57], suggesting that accurate quantification is possible.

Further testing on preservation methods is essential in terms of the effect on the quantitative accuracy over time, both those for microscopy and multiplex PCR techniques. The maximum storage time in potassium dichromate is currently undetermined, however reports have suggested a one month storage period in potassium dichromate for *Giardia* for optimal detection [33]. Recent reports on formalin preserved samples suggest a 15 day storage window in which microscopy can be completed to gain accurate quantitative hookworm data, with additional decline in the ability to detect parasites after one month [52]. Microscopy on samples in this study was not reliably performed within this optimum time period; potentially resulting in the lower prevalence and intensity data for hookworms.

The main limitation of multiplex PCR in national control programmes is the capacity to implement it in parasite endemic low-resource settings, with the current need to send samples to well equipped labs for analysis. Microscopy based techniques can however, be undertaken with less resources and more affordable equipment. The material cost of processing samples and running both multiplex PCRs was estimated as AU$12.37 per sample (AU$6.05 per extraction; AU$3.16 per multiplex PCR). This also represents a limitation when compared to flotation based microscopy, costing just AU$1 per sample (labour not included). The trade off of the additional costs and the inability for onsite analysis compared to the higher sensitivity and ability to detect multiple infections as well as the more accurate intensity data is an issue for consideration in design of epidemiologic studies and for clinical trials of effectiveness of interventions.

The use of multiplex real-time PCR for intestinal parasite diagnosis has proved to be more sensitive, and is more likely to detect mixed parasite infections than standard microscopy techniques. The real benefit of multiplex PCR is in its ability to more accurately determine infection intensity and the potential to report results in more understandable ‘EPG’ terms, which will prove to be inherently more useful in determining the success of de-worming and intervention trials.

## Supporting Information

S1 Methods Method A: Microscopy. Method B: DNA Extraction. Method C: Multiplex PCR Optimization. (DOCX)

S1 Fig EHV Extraction/PCR Control Example Real time PCR Results. Average EHV Ct-values for each of the 37 field DNA samples compared within PCR runs to EHV-only control sample. Major outlier amplification from each run indicated in red where repeat extraction is required. Minor deviations outside 10–90^th^ percentile interval also considered for repeat extraction or repeat PCR. The specific source of the problem (PCR or extraction) was determined through EHV data comparison between the quantitative multiplex and semi-quantitative multiplex.
(TIF)

S2 Fig Intensity of Infection for *Ascaris* positive samples as determined by PCR with calibration curve. The *Ascaris* Ct-EPG interpolation formula was used on 131 Timor-Leste *Ascaris* positive samples that were within the required Ct range (15.5–31.5) to allow adequate prediction of EPG from Ct value using calibration curve (EPG = 10^−0.275^*^Ct +9.622^). Multiplex real-time PCR duplicates were each individually calculated with mean and SD for each sample. **(A)** Calibration curve example—converting PCR Ct-values to EPG **(B)** The infection intensity range for these 131 samples within acceptable calibration curve range.
(TIF)

S1 Dataset (XLSX)
