# Supplementary material for: Application of a Multiplex Quantitative PCR to Assess Prevalence and Intensity Of Intestinal Parasite Infections in a Controlled Clinical Trial
Source: PLoS Negl Trop Dis. 2016 Jan 28;10(1):e0004380. doi: 10.1371/journal.pntd.0004380 (PMC4731196; doi:10.1371/journal.pntd.0004380)
Supplement: S1 Methods — (DOCX) [file pntd.0004380.s001.docx]

**S1 Methods**

**Method A: Microscopy**

Sodium nitrate flotation for STH ([1](#_ENREF_1)) involved removal of formalin followed by mixing two grams of each stool sample with four times its volume of distilled water. This suspension was strained through a dual layer surgical gauze lined funnel into a 10 ml centrifuge tube followed by centrifugation for two minutes at 3,000g. Supernatant was poured off leaving 250 mg fecal pellet to which sodium nitrate solution (S.G. 1.20) was added to the rim of the tube forming a positive meniscus, and a 22mm x 22mm cover slip placed on top. After 10 min the coverslip was removed and placed onto a microscopy slide. The entire slide was examined and total number of eggs on the cover slip counted, with total number multiplied by four to obtain total EPG.

Zinc sulphate centrifugal flotation for protozoa ([2](#_ENREF_2)) involved removal of formalin, followed by mixing ~ 1 g faeces with 9 ml water in a 10 ml centrifuge tube followed by 2000 g centrifugation for 3 min, with pellet discarded. The pellet was resuspended in 9 ml of ZnSO_4_ (S.G. 1.18) and centrifuged at 2000 g for 3 min. A wire loop was used to remove a small volume of faecal suspension to place on a slide. This was repeated four times before a cover slip placed on top and microscopic examination at 100 x and 400 x magnification.

1. Inpankaew T, Traub R, Thompson R, Sukthana Y. Canine parasitic zoonoses in Bangkok temples. Southeast Asian J Trop Med Public Health. 2007;38:247 - 55.

2. Traub RJ, Inpankaew T, Reid SA, Sutthikornchai C, Sukthana Y, Robertson ID, et al. Transmission cycles of *Giardia duodenalis* in dogs and humans in Temple communities in Bangkok--a critical evaluation of its prevalence using three diagnostic tests in the field in the absence of a gold standard. Acta Trop. 2009;111(2):125-32.

**Method B: DNA Extraction**

The Powersoil DNA Isolation Kit (Mo Bio, Carlsbad, CA USA) was used for DNA extraction with minor modifications. Briefly, Zirconia/Silica 0.5 mm beads (Daintree Scientific, St.Helens, Tasmania AUS) were used in place of supplied beads. Each extraction used 370 µL of the Powerbead solution and 0.2 g faeces. The Precellys®24 Lyser/Homogeniser (Bertin Technologies, Montigny-le-Bretonneux, France) (setting “6500 1x60 015”) was used in a one minute homogenization step in place of vortexing. Further alterations to kit protocol included: processing the entire supernatant through each step to avoid loss of material, reducing the volume of Solution C4 to 1 ml, and an additional standard dry filter centrifugation step following Solution C5 removal. DNA was eluted from the column in a 100 µL of buffer and stored at -20˚C prior to PCR. An EHV-only control was included in each batch processed as a comparison.

**Method C: Multiplex PCR Optimisation**

Minor modifications were made to previously published protocols ([3](#_ENREF_3)). Briefly, the amplification reaction mixture consisted of Hotstar Taq mastermix (Qiagen), 5mM MgCl_2_ (Bioline(Aust)), optimized primer and probes as listed in Table 1 and 2 µL of template DNA in a total volume of 20 µL. For the quantitative STH multiplex PCR the DNA amplification was performed using the following conditions: 15 minutes at 95 ˚C followed by 40 cycles of 95˚C for 9 seconds and 60˚C for 60 seconds. The semi-quantitative protozoa and *S. stercoralis* multiplex PCR was performed using the following conditions: 15 minutes at 95 ˚C followed by 40 cycles of 95˚C for 30 seconds, 55˚C for 30 seconds and 72˚C for 30 seconds. The cycling threshold held constant at 0.016 units for each multiplex PCR. Assay optimisation was initially undertaken for each target in conventional singleplex PCR with positive control genomic DNA as template (*A. duodenale*, *N. americanus* and *Ascaris lumbricoides*; *E. histolytica*, *G. duodenalis*, and *C. parvum,* *Strongyloides ratti* and *T. vulpis*). Samples were visualised on a 1.5 % agarose gel in Tris Acetate buffer using SYBR safe^®^ Nucleic Acid Gel Stain (Life Technologies, Invitrogen, Eugene, USA). PCR products were purified using the High Pure PCR Purification kit (Roche, Basel, Switzerland), and verified by sub-cloning using the pGEM vector system (Promega Corporation, Madison WI USA) to produce plasmid controls. Plasmid pools were made containing each plasmid for each multiplex diluted to similar concentrations (Cycle threshold (Ct) ~10), and used to make the standard curves incorporated in each multiplex reaction as a PCR control and as a reaction efficiency indicator.

The concentrations for each primer pair within each multiplex reaction were optimised in singleplex PCR by primer limiting experiments using plasmid controls of each PCR. Ct-value comparison between multiplex and singleplex PCR assays were also performed, with concentrations selected for greatest sensitivity without detrimentally affecting consecutive PCR reactions. Further optimisations were also performed to compare the ability of the multiplex PCR to detect mixed infections with dilutions of target organisms tested across a range of background DNA. The fluorescence threshold was set at 10% for *N. americanus*, *Ancylostoma* spp., and *Cryptosporidium* spp.; 20% for *G. duodenalis*; and 30% for *Ascaris* spp., *T. trichiura*, *E. histolytica*, *Strongyloides* spp., and the EHV control.

The EHV Ct-value was monitored for each sample, with values more than two cycles higher than the expected Ct-value considered unsuccessful, with repeat extraction required. The expected EHV Ct-value was determined by comparison to the EHV-only control and surrounding samples.

1. Inpankaew T, Traub R, Thompson R, Sukthana Y. Canine parasitic zoonoses in Bangkok temples. Southeast Asian J Trop Med Public Health. 2007;38:247 - 55.

2. Traub RJ, Inpankaew T, Reid SA, Sutthikornchai C, Sukthana Y, Robertson ID, et al. Transmission cycles of *Giardia duodenalis* in dogs and humans in Temple communities in Bangkok--a critical evaluation of its prevalence using three diagnostic tests in the field in the absence of a gold standard. Acta Trop. 2009;111(2):125-32.

3. Basuni M, Muhi J, Othman N, Verweij JJ, Ahmad M, Miswan N, et al. A pentaplex real-time polymerase chain reaction assay for detection of four species of soil-transmitted helminths. Am J Trop Med Hyg. 2011;84(2):338-43.
