# Supplementary figures and images for: Application of a Multiplex Quantitative PCR to Assess Prevalence and Intensity Of Intestinal Parasite Infections in a Controlled Clinical Trial
Source: PLoS Negl Trop Dis. 2016 Jan 28;10(1):e0004380. doi: 10.1371/journal.pntd.0004380 (PMC4731196; doi:10.1371/journal.pntd.0004380)

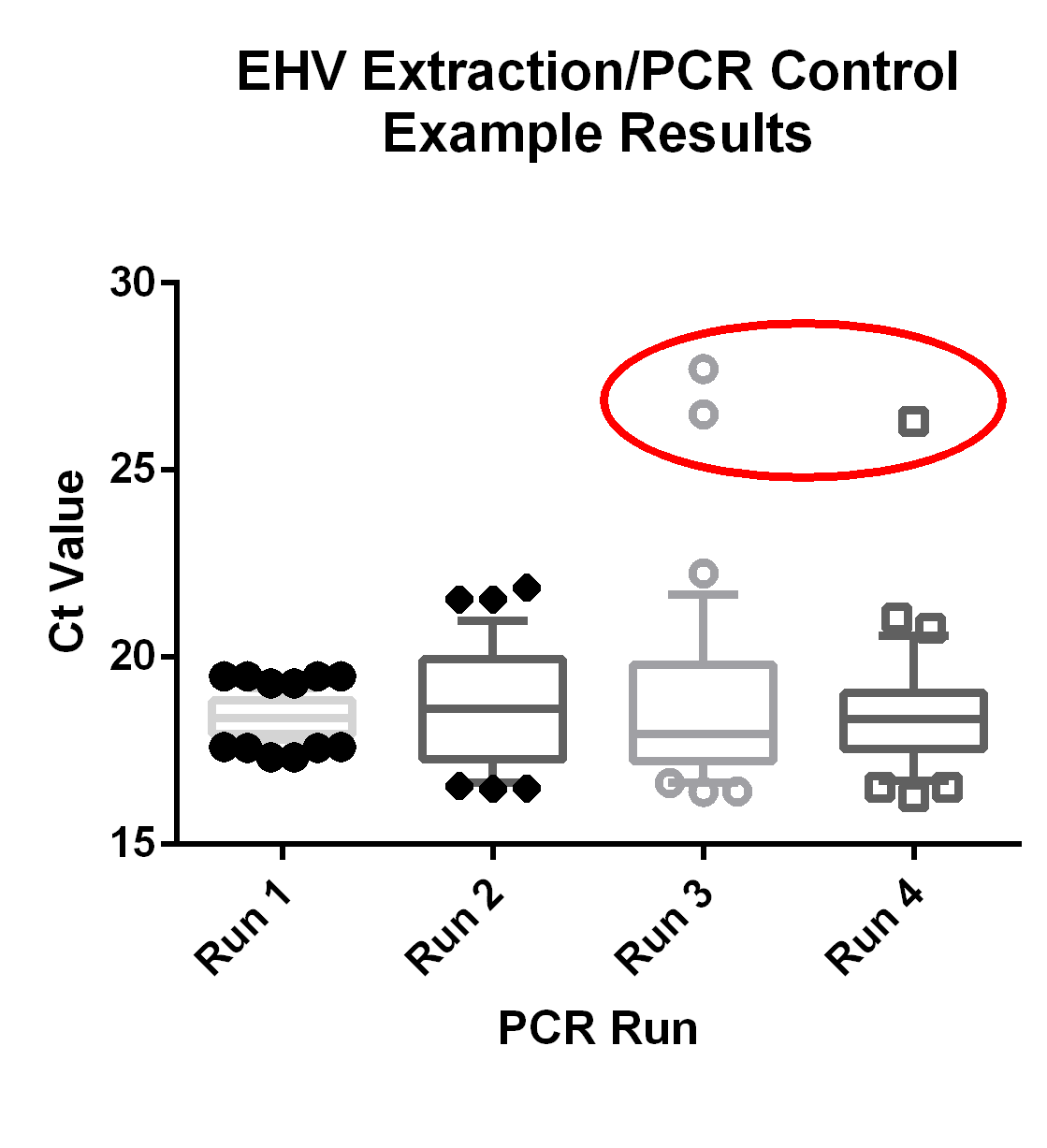

Supplement: S1 Fig — Average EHV Ct-values for each of the 37 field DNA samples compared within PCR runs to EHV-only control sample. Major outlier amplification from each run indicated in red where repeat extraction is required. Minor deviations outside 10–90th percentile interval also considered for repeat extraction or repeat PCR. The specific source of the problem (PCR or extraction) was determined through EHV data comparison between the quantitative multiplex and semi-quantitative multiplex. (TIF) [file pntd.0004380.s002.tif]

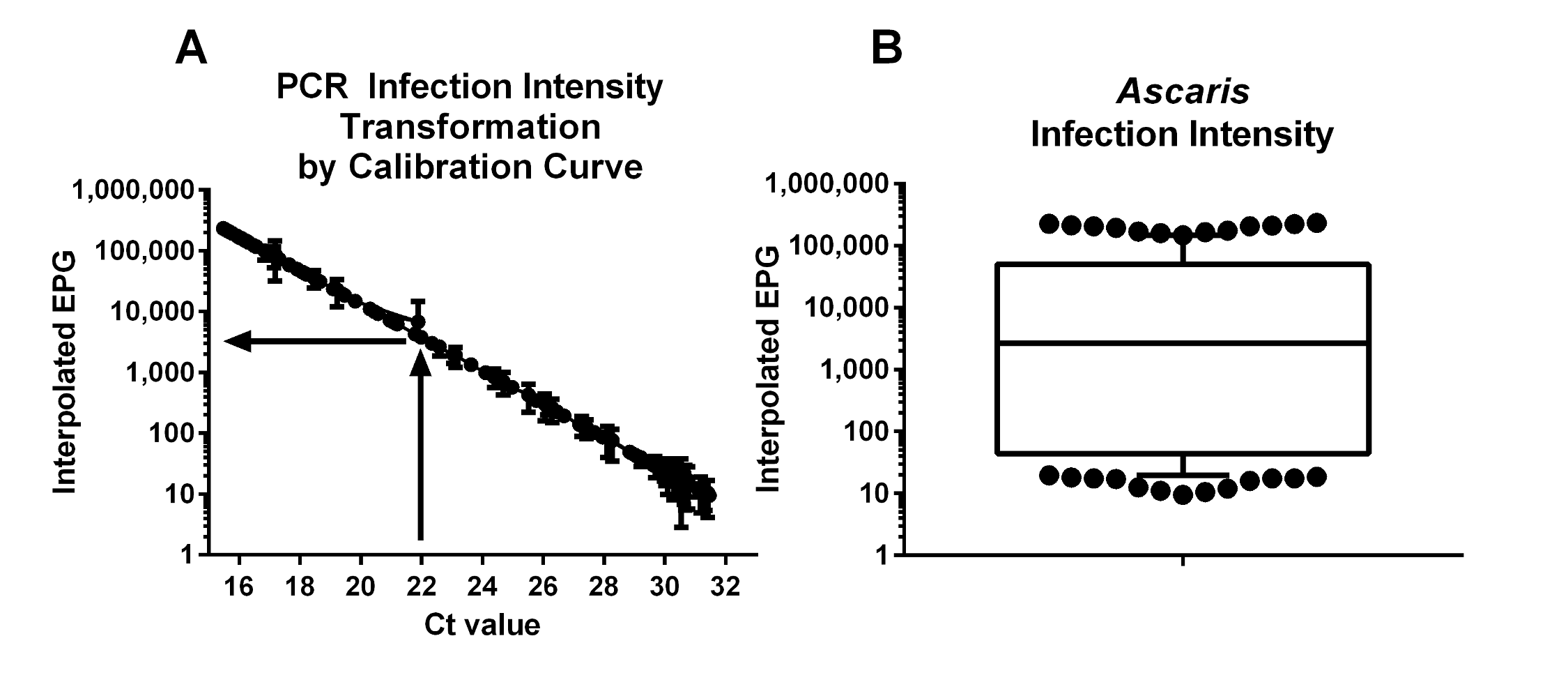

Supplement: S2 Fig — The Ascaris Ct-EPG interpolation formula was used on 131 Timor-Leste Ascaris positive samples that were within the required Ct range (15.5–31.5) to allow adequate prediction of EPG from Ct value using calibration curve (EPG = 10−0.275*Ct +9.622). Multiplex real-time PCR duplicates were each individually calculated with mean and SD for each sample. (A) Calibration curve example—converting PCR Ct-values to EPG (B) The infection intensity range for these 131 samples within acceptable calibration curve range. (TIF) [file pntd.0004380.s003.tif]
